# Development of an Epoxy Matrix Hybrid Composite with Astrocaryum Aculeatum (Tucumã) Endocarp and Kaolin from the Amazonas State in Brazil

**DOI:** 10.3390/polym15112532

**Published:** 2023-05-31

**Authors:** Antonio Claudio Kieling, José Costa de Macedo Neto, Gilberto Garcia del Pino, Ricardo da Silva Barboza, Francisco Rolando Valenzuela Diáz, José Luis Valin Rivera, Meylí Valin Fernández, Cristobal Galleguillos Ketterer, Alvaro González Ortega, Roberto Iquilio Abarzúa

**Affiliations:** 1 Department of Mechanical Engineering, State University of Amazonas, Manaus 69850-020, Brazil; gpino@uea.edu.br; 2 Department of Materials Engineering, State University of Amazonas, Manaus 69850-020, Brazil; jmacedo@uea.edu.br; 3 Computer Engineering Department, State University of Amazonas, Manaus 69850-020, Brazil; rsbarboza@uea.edu.br; 4 Department of Materials Engineering and Metallurgy, University of São Paulo, São Paulo 05508-030, Brazil; frrvdiaz@usp.br; 5 Escuela de Ingeniería Mecánica, Pontificia Universidad Católica de Valparaíso, Valparaíso 2340025, Chile; jose.valin@pucv.cl (J.L.V.R.); cristobal.galleguillos@pucv.cl (C.G.K.); alvaro.gonzalez.o@pucv.cl (A.G.O.); roberto.iquilio@pucv.cl (R.I.A.); 6 Department of Mechanical Engineering (DIM), Faculty of Engineering (FI), University of Concepción, Concepción 4030000, Chile; mvalin@udec.cl

**Keywords:** composite materials, tucumã, kaolin, epoxy resin

## Abstract

Composites with natural lignocellulosic fillers are being cited as a viable and sustainable alternative to conventional materials, as they combine lower costs with lower weight. In many tropical countries, such as Brazil, there is a considerable amount of lignocellulosic waste that is improperly discarded, which causes pollution of the environment. The Amazon region has huge deposits of clay silicate materials in the Negro River basin, such as kaolin, which can be used as fillers in polymeric composite materials. This work investigates a new composite material (ETK) made of epoxy resin (ER), powdered tucumã endocarp (PTE), and kaolin (K), without coupling agents, with the aim of producing a composite with lower environmental impact. The ETK samples, totaling 25 different compositions, were prepared by cold molding. Characterizations of the samples were performed using a scanning electron microscope (SEM) and a Fourier-transform infrared spectrometer (FTIR). In addition, the mechanical properties were determined via tensile, compressive, three-point flexural and impact tests. The FTIR and SEM results showed an interaction between ER, PTE, and K, and the incorporation of PTE and K reduced the mechanical properties of the ETK samples. Nonetheless, these composites can be considered potential materials to be used for sustainable engineering applications in which high mechanical strength is not a main requirement of the material.

## 1. Introduction

With the development of new production technologies that demonstrate significant increases in quality and reductions in aggressive waste into the environment, it has become important to use new materials and techniques to improve the properties of such materials [1,2]. Among these new materials are composite materials that first emerged in ancient Egypt and Mesopotamia as mixtures of mud and straw, as well as combinations of other plant fibers that have been found by archaeologists in ancient burial chambers. The current era of composite materials began with the discovery of plastic and its numerous applications [3].

Due to their ease of production and their low weight and flexibility, polymers have been applied in various solutions in a wide range of industrial sectors [4]. Among the various forms of polymeric materials that are now on the market, epoxy resins stand out, and their particular characteristic of thermosetting is of industrial interest due to their properties of chemical resistance, electrical insulation, thermal and mechanical characteristics, and low contraction during their curing. With their huge commercial production for applications in, for example, the automotive and aerospace industries, the global epoxy market reached USD 26 billion in 2019, with an expected increase from 2020 to 2028 of a compound annual growth rate of 6.2% [5,6,7,8].

Different fibers—mainly synthetic fibers such as glass fiber, carbon fiber, and aramid—are used for composite materials, and clays are being used to reinforce these materials. Recently, the addition of clay nanoparticles has attracted attention in academic and industrial circles due to improvements in mechanical and thermal properties [9]. Clays are abundant materials in nature and, due to the combination of low cost and the low environmental impact involved in obtaining them, the study and development of applications of their nanoparticles has been widespread [10]. The State of Amazonas, which is the largest state in Brazil, is rich in deposits of clay minerals, principally in the geological formations of the Solimões (upper Miocene period) and Içá (Pleistocene period), with a predominance of kaolinite (kaolin) deposits [11].

In composite studies, bentonite nanoparticles with maleic anhydride added to polymers at levels of up to 5% mass provided an increase in tensile strength and modulus of elasticity of up to 35%, as demonstrated by Venkatesan et al. [12] and Rao and Sankar [13]. The addition of clay nanoparticles has resulted in significant improvements in mechanical properties, UV resistance, and anti-bubble characteristics in different types of composites with plant fibers, as shown by Deka et al. [14]. The addition of organophilic bentonite nanoparticles in the order of 2.5%, 5%, and 10% by weight in polyester resulted in a composite with higher tensile, flexural, and impact strength, as demonstrated by Garcia et al. [15].

The application of lignocellulosic fibers as reinforcements in composite materials has found increasing use in recent years, replacing synthetic fibers such as glass, carbon, and aramid fibers, due to the characteristics of natural fibers, such as their low cost, low density, high specific modulus, economic and environmental advantages, biodegradability, abundance, and many technical qualities [15,16,17]. These advantages place lignocellulosic fiber composites among the high-performance composites, with economic and environmental advantages [18,19]. Although completely synthetic composites dominate the automobile, aircraft, sporting goods, and infrastructure sectors [8,9,10], they have significant disadvantages, such as high input costs, high costs of production, non-recyclability, toxicity, and non-biodegradability [20,21,22].

The tucumã (Astrocaryum aculeatum) is a palm tree that is native to the Amazon, and the pulp of its fruit is consumed in several dishes in the cuisine of the Amazon. It has high lipid, energy, and vitamin A content, and its content of beta-carotene is considered medium. Its core is used in local crafts, although 92% is discarded as trash without reuse—discards that could yield up to 30 tons of woody endocarp on a monthly basis, as demonstrated by Kieling et al. [23].

Composites with thermoplastic polymer and powdered tucumã endocarp were developed by the addition of 10%, 20%, 30%, 40%, and 50%, by weight in a recycled polypropylene matrix. Additionally, endocarps of other fruits, such as coconut, can be used for applications that are similar to those of the endocarp of tucumã, as shown by Kieling et al. [20].

In this work, we discuss the development of a composite produced with powdered tucumã endocarp (PTE) and kaolin clay (K) in the epoxy resin (ER) matrix and provide an analysis of that development. The tucumã fruit is highly consumed in the State of Amazonas and there are large amounts of endocarpium that are not used. Such amounts of unused endocarpium could be utilized in compost. In addition, there are large reserves of kaolin in the State of Amazonas which, together with tucumã, could be used in composites to reduce the cost of materials. To carry out this study, different techniques for characterizing materials were used, as well as tensile, bending and impact tests.

## 2. Materials and Methods

**Figure 1:**
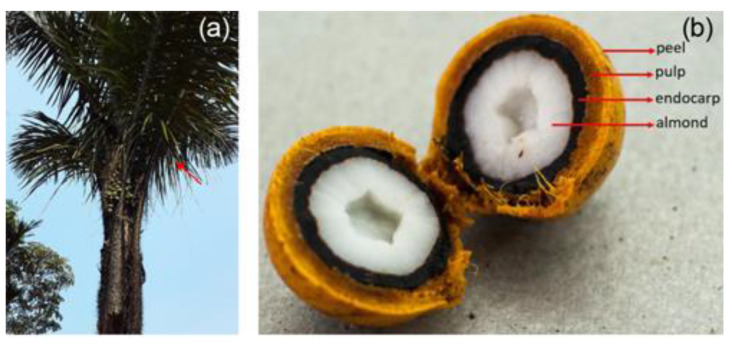
Characteristics of the tucumã: (**a**) tucumã palm and (**b**) fruit of the tucumã palm [20].

The manufacture of the composite began at the Manaus Moderna market with the acquisition of 250 tucumã cores with an average diameter of 34.28 ± 4.84 mm and an average mass of 22.08 ± 2.63 g (measurement with a Mitutoyo digital caliper, precision 0.05 mm, and a Prix 3400 analytical balance, precision 0.001 g). The tucumã endocarp was initially processed by breaking the cores with the help of a sledgehammer, by which the woody endocarp was separated from the fleshy inner, taking care to break it into small parts. Afterwards, the material was ground in knife mill (Marconi, 200 mesh). The material was then dried at 60 °C in a vacuum oven (Quimis) for four hours and the powder was stored in a container inside a dryer for further use. Figure 1 shows details of the tucumã fruit, where the endocarp that was used in this work can be seen.

The kaolin for this study (kaolin-AM, white in color) was obtained in the city of Careiro, Amazonas (3°49′10.0″ S 60°21′40.4″ W). Initially, the kaolin was dried in an oven (Quimis) with air circulation for 24 h at a temperature of 105 °C. Subsequently, the material passed through a sieve tower with a final mesh of 200.

Transparent epoxy resin (2001, Redelease do Brasil) with hardener (3154, Redelease do Brasil) was used in the ratio of 1:2 (resin to hardener) by mass/weight. In addition, to avoid the formation of bubbles as much as possible, the anti-bubble additive (Siladit 53, Redelease do Brasil) for epoxy resins was used, at a proportion of 0.5% of the total weight of the formulation.

**Figure 2:**
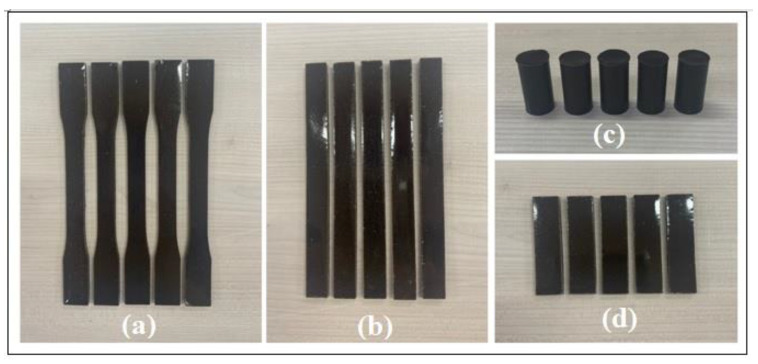
ER + PTE + kaolin composite test samples for the following tests: (**a**) tensile, (**b**) flexural, (**c**) compression, and (**d**) Izod impact.

The samples were produced considering variations with and without the clay and also with and without the powdered tucumã endocarp, and totaled 25 types of samples, as shown in Table 1. The name of the samples was considered as epoxy/tucumã/kaolin (ETK). The following sequence was used in the formulation and manipulation of the composite material: (a) resin, (b) hardener, (c) additive, (d) powder, and (e) kaolin. The materials were deposited in the sequence indicated in a transparent polypropylene cup, and the mixture was homogenized for 5 min with a bamboo spatula (rod). Then, the material was deposited in metal molds up to the thickness limit. A level meter (Mitutoyo) with a resolution of 0.1° was used to level the molds on the workbench. The resin was left to cure for 24 h at a temperature greater than 18 °C. After the curing time, the samples were removed from the molds. Figure 2 shows the specimens manufactured using this methodology.

**Figure 3:**
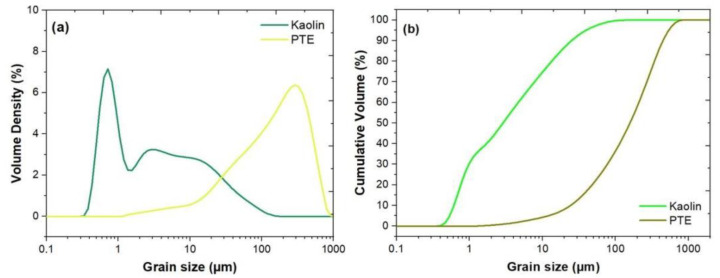
Distribution of (**a**) individual granulometric and (**b**) cumulative volume of kaolin and powdered tucumã (PTE) [26].

The PTE and kaolin were analyzed for particle size (Mastersizer 2000, Malvern Instruments, resolution from 0.02 μm to 2000 μm) and their cumulative volumetric dispersion, according to ASTM d4464-14 [24]. The objectives of carrying out the characterization of the particle size were as follows: first, to verify whether the granulometry presented in this work coincided with the granulometry presented in previous works such as [25] and second, to use it as a reference base to analyze, in future works, the influence of particle size on the mechanical properties of the composite. In the sample preparation, distilled water was used as dispersant medium, 1.56 refractive index, 0.1 absorption, 1750 rpm stirring and pumping. The previously dispersed samples were placed in the vat to obtain the readings. For the homogenization of the samples, it was necessary to treat them with 1 min of ultrasound following the equipment PLANATC, CBU100/3LDG. The results obtained can be seen in the graph of Figure 3.

**Figure 4:**
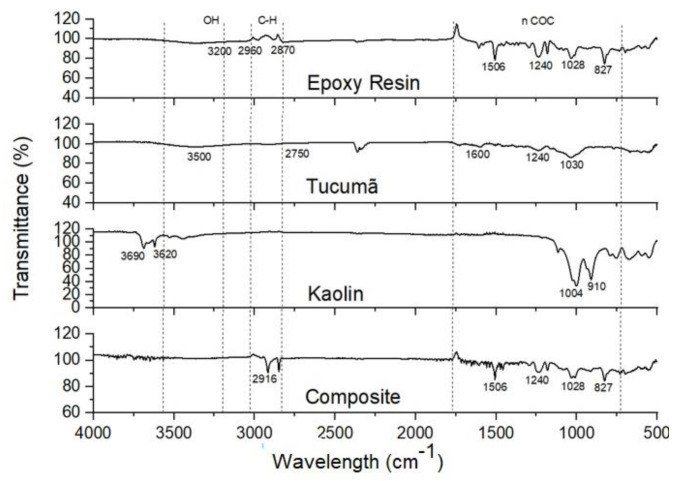
FTIR spectrum for ER, PTE, kaolin from the State of Amazonas, and the resulting composite ETK13 [26].

With the objective of knowing the different chemical groups that were present in the tucumã powder, in the kaolin, and then in the interaction of these components in the composite, a characterization of the composite in its different combinations was performed using a Fourier-transform infrared spectrometer (FTIR) (Shimazdu, IRAffinity-1S) in samples ranging from 10 to 20 mg, as recommended by the ASTM E-1252-98 standard [27]. The analyses were performed on the attenuated total reflectance (ATR) module (%T as a function of the wave number in cm^−1^). The spectra were obtained in the mid-infrared spectroscopy (MIR) region, in the range of 4000 to 500 cm^−1^. The results obtained can be seen in the graph provided in Figure 4.

The percentage of the chemical components of the vegetable fibers was determined following the Van Soest methodology [28], which uses an acid-type detergent, cetyl trimethyl ammonium bromide (CTAB), to determine the percentage of ADF (fiber + lignin), lignin and cellulose of the vegetable fibers. For this, four samples were elaborated, using 27.19 mL of concentrated H_2_SO_4_ for 1 L of distilled water and 720 mL of concentrated H_2_SO_4_ for 280 mL of distilled water, and they were dissolved in 50 g of CTAB in 5 L of 0.5 M H_2_SO_4_. First, 1 g (weight 1) of plant material was weighed in a 250 mL Erlenmeyer flask. Next, 100 mL of CTAB and drops of octan-2-ol were added as antifoam, and then it was heated for 1 h at a temperature of 80 °C to 90 °C. To determine the percentage of ADF, the extract was filtered under hot water, pre-ignition at 550 °C, and weighed (weight 2). The residue was washed three times with 50 mL of distilled water at approximately 95 °C to 100 °C and then with acetone, until the acetone was crystalline. Suction was imposed until the sample was dry, finishing the drying in an oven at 105 °C for 2 h to be weighed later (weight 3). The ADF percentage was determined by Equation (1).

(1) $$\%ADF=\frac{\left(weight3-weight2\right)\cdot 100}{weight1}$$

To determine the percentage of cellulose, the H_2_SO_4_ solution (cold) was added, homogenizing it with a glass rod. Instead of draining naturally, the same quantity of H_2_SO_4_ was placed again, homogenizing it again. After 3 h, hot water was added, and the acid was removed by suction. Next, the sample was washed using acetone, dried first by suction, and then in an oven at 105 °C for 2 h, to be weighed (weight 4). The cellulose percentage was determined by Equation (2).

(2) $$\%Celulose=\frac{\left(weight3-weight4\right)\cdot 100}{weight1}$$

To determine the lignin, the sample was subjected to 550 °C in a muffle furnace for 2 h, then cooled and weighed (weight 5). The lignin percentage was determined by Equation (3).

(3) $$\%Lignin=\frac{\left(weight4-weight5\right)\cdot 100}{weight1}$$

**Figure 5:**
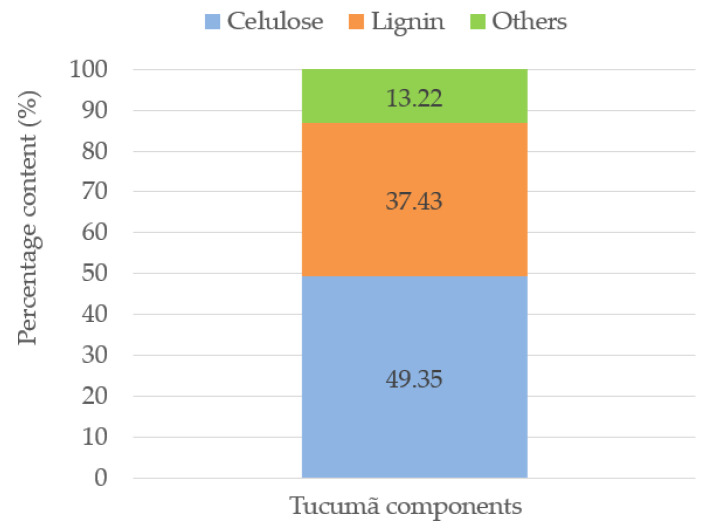
Components of vegetable fibers.

The results obtained of cellulose and lignin percentage are shown in Figure 5.

**Figure 6:**
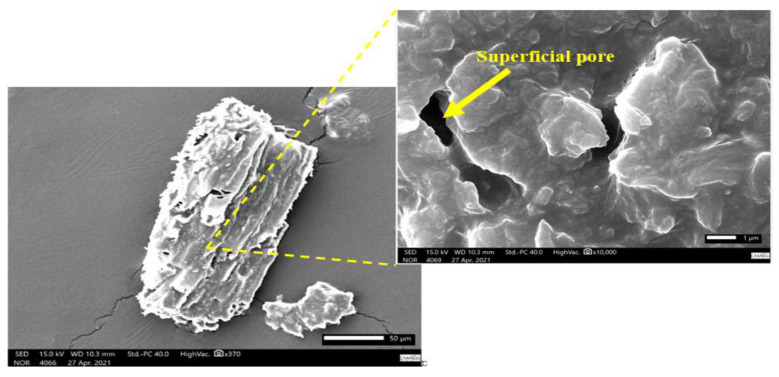
SEM micrograph of PTE before tensile test.

**Figure 7:**
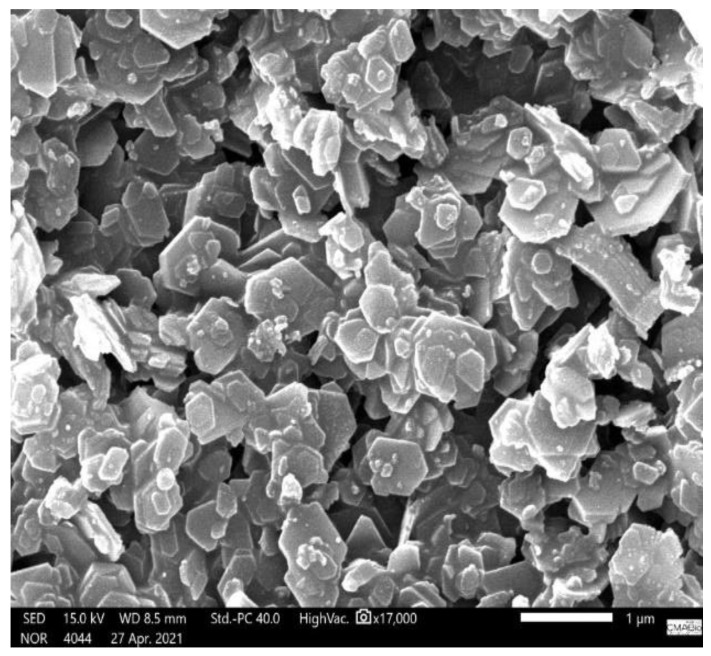
SEM micrograph of kaolin before traction test [26].

**Figure 8:**
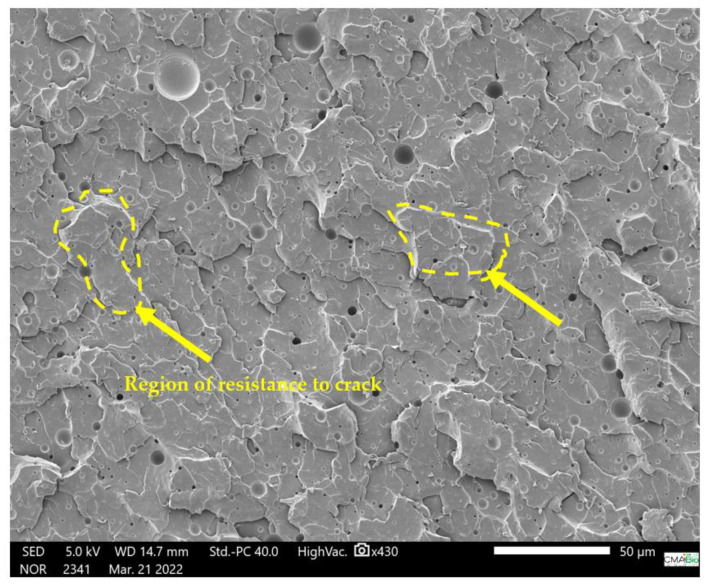
SEM micrograph of ER + 2% kaolin (ETK2) in fracture region after tensile test.

**Figure 9:**
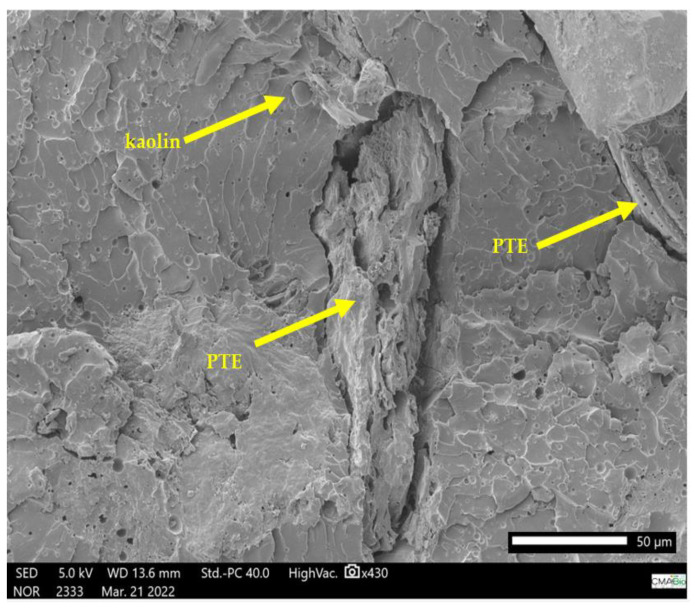
SEM micrograph of ER + 10% PTE + 4% kaolin (ETK8) in fracture region after tensile test.

**Figure 10:**
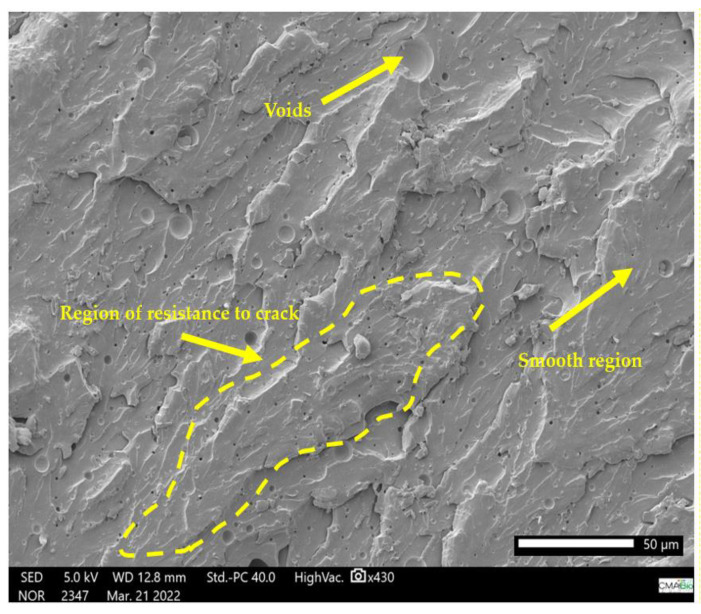
SEM micrograph 100% ER (ETK1) in fracture region after tensile test.

**Figure 11:**
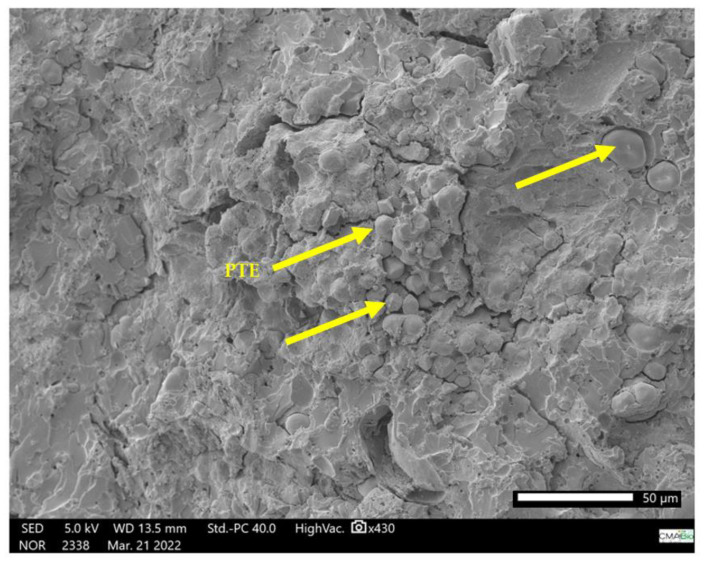
SEM micrograph of ER + 40% PTE + 2% kaolin (ETK22) in fracture region after tensile test.

Subsequently, scanning electron microscope (SEM) micrographs of the kaolin and tucumã endocarp powder reinforcement were performed separately and on the composite to study the distribution of these components within the composite. Initially, gold metallization was performed in all samples to increase the conduction of electrons and allow for a better image. Then, using a scanning electron microscope (JEOL JSM-IT500HR), the voltage acceleration was adjusted to 15 kV with a secondary electron beam at varying magnifications. The ER and ETKs samples used in the analyses were obtained after the performance of the tensile test, and their fracture regions were preserved; the PTE and kaolin samples were also analyzed. The reference standard employed was ASTM E986-04 [29] and the results obtained are shown in Figures 6–11.

Finally, to determine the mechanical properties of the hybrid composite as a function of the amount of powdered tucumã endocarp and kaolin, mechanical tests of traction, compression, flexion, and impact were carried out on the specimens made in molds, as shown in Figure 2. For the tensile, flexural, and compression tests, a universal test machine (Instron 5984) was used, which was equipped with a 150 kN load cell with a displacement speed in the order of 10 mm min^−1^. A set of five samples was tested for each composition. The tensile test was carried out according to the reference standard ASTM D638-14 [30], considering the type I test sample with a size of 165 × 19 × 3.2 mm.

The flexural test was performed in accordance with the ASTM D790-03 standard [31]. In the performance of the test, procedure A was adopted using the three-point method, considering a specimen with dimensions of 127 × 12.7 × 3.2 mm.

The compression test was carried out according to the reference standard ASTM D695-2A [32], considering a sample with a length of 25.4 mm and a diameter of 12.7 mm.

The reference standard for the Izod impact test, ASTM D256-04 [33], which is recommended for polymeric materials and their derivatives, was applied to three samples for each composition. The impact rupture energy of the polymeric material is quantified in the form of joules per meter (J/m). Test type A of the standard was chosen for use with samples of 63.5 × 12.5 × 104 3.2 mm with notches at 45° in the middle. A pendulum impact testing machine on IT504 plastics (Tinius Olsen, Horsham, PA, USA) was used.

## 3. Results

### 3.1. Particle Size Characterization

In Figure 3a, it can be observed that the granulometric curve of the powdered tucumã presents an approximately normal distribution, while the kaolin presents a bimodal distribution with deviation to the left. In the particle size range of 0.6 µm to 290 µm for kaolin and powdered tucumã, respectively, there are significant peaks, with volume ratios of 6.7 to 6.4. For kaolin, the peak volume ratio was larger, and the particle size was finer than in the powdered tucumã. The curves of granulometric distribution by accumulated volume are presented in Figure 3b. For kaolin, the effective diameter (d10) was 0.61 μm, and 10% of the particles had a diameter less than this value [31], while for the powdered tucumã it was 24.5 μm. It can be seen that, for kaolin, 43% of the particles were below 2 μm and 87% were below 20 μm, while for tucumã powder, 8% were below 20 μm, 37% were below 100 μm, and 8% were below 330 μm. The powder, which has a very fine granulation, has a degraded appearance and, therefore, it was preferable to work with the granulation presented above.

### 3.2. FTIR Characterization

Figure 4 shows the results of the FTIR assay for the ER, PTE, kaolin, and the resulting composite ETKs. Absorptions were found in the middle part of the spectrum for ER, which consists of epoxy and hydrocarbon groups. Vibrational regions of C-H elongation were observed between 2870 cm^−1^ and 3025 cm^−1^, with hydroxyl groups vibrating at 3200 cm^−1^ and epoxy functional groups at around 1000 cm^−1^, with significant peaks between 827 cm^−1^ and 1506 cm^−1^, which were similar to those mentioned by Zhang et al. [34].

The PTE exhibited molecular group vibrations in the range of 3500 cm^−1^, referring to O-H stretch that was due to existing moisture. In the region of 2750 cm^−1^, O-H binding bands were identified. The addition of powdered tucumã tended to decrease the presence of benzene rings in the molecular structure, thus explaining the potential loss of mechanical properties, which was corroborated by the increase of peaks in the region of 1500 cm^−1^ to 1600 cm^−1^ of the aromatic groups; this is also mentioned by Otieno et al. [35] and Kieling et al. (2023) [26].

In the region of 3620 cm^−1^ to 3680 cm^−1^, kaolin showed O-H stretching due to the presence of moisture, and at 1120 cm^−1^ longitudinal Si-O stretching, between 1000 cm^−1^ and 1030 cm^−1^ Si-O stretching due to the presence of quartz, O-H folding at 911 cm^−1^ from internal hydroxyl, Si-O folding at 753 cm^−1^, perpendicular stretching at 755 cm^−1^, Si-O-Si folding at 677 cm^−1^, Al-O-Si folding at 556 cm^−1^, and Si-O folding at 427 cm^−1^, which were similar to what was indicated by Deju et al. [36].

The spectrum of ETK13 indicated combined characteristics of ER + PTE + kaolin in the regions between 820 cm^−1^ to 1200 cm^−1^, 1400 cm^−1^ to 1600 cm^−1^, and 3580 cm^−1^ to 3800 cm^−1^, and it can be concluded that there is an interaction between the elements in the formation of the composite.

### 3.3. Percentage of Chemical Components of Vegetable Fibers

As indicated in the section on materials and methods, the components of the vegetable fiber were determined using the Van Soest methodology [28], using Equations (1)–(3). The results obtained are shown in Figure 5, where tucumã is composed of 49.35% of cellulose, 37.43% of lignin, and 13.22% of other components.

### 3.4. SEM Characterization of the ER, Kaolin, and ETKS

Figure 6, Figure 7, Figure 8, Figure 9, Figure 10 and Figure 11 show the SEM images generated in isolation for the powdered tucumã, kaolin, and epoxy resin, as well as the images of the combinations of these elements. Isolated images of clay and PTE show their morphologies and images of composites show particle distribution and interaction with the epoxy matrix. The surface of the PTE (Figure 6) appears to be rough, prismatic, and irregular with pores, similar to that seen by Kieling et al. [20].

The kaolin (Figure 7) contains its characteristic pseudo-hexagonal structure in the form of lamellar layers, as evidenced in the study of Otieno et al. [35].

The ER + 2% kaolin (ETK2) composite (Figure 8) has small particles of scattered kaolin grains in the ER matrix.

The ER + PTE + kaolin composites (ETK8) (Figure 9) present more evident regions of rupture, with a dispersion of the kaolin and PTE grains in the ER structure, in which the granular forms of the added fillers are not clearly evident.

This characterizes the intermolecular interaction resulting from these elements added to the ER and is corroborated by the interaction of the benzene rings observed in the FTIR assay and by the effect of the addition of PTE in the ER, combined with the addition of kaolin. The ER (ETK1) (Figure 10) has a structure of the fracture in the form of a smooth, region of resistance to crack propagation that led to brittle failure and voids [33].

The ER (ETK1) (Figure 10) has a structure in the form of overlapping layers due to the cold molding process. The ER + PTE + kaolin composites (ETK8 and ETK22) (Figure 9 and Figure 11) present more evident regions of rupture, with a dispersion of the kaolin and PTE grains in the ER structure, in which the granular forms of the added fillers are not clearly evident.

This characterizes the intermolecular interaction resulting from these elements added to the ER and is corroborated by the interaction of the benzene rings observed in the FTIR assay and by the effect of the addition of PTE in the ER, combined with the addition of kaolin. Microbubbles are formed in the samples (Figure 7, Figure 8, Figure 9 and Figure 10) even with the addition of the anti-bubble additive as recommended by the manufacturer of the ER, which also contributes to a reduction in the mechanical strength of the resulting composites.

### 3.5. Mechanical Properties of the Composite

**Figure 12:**
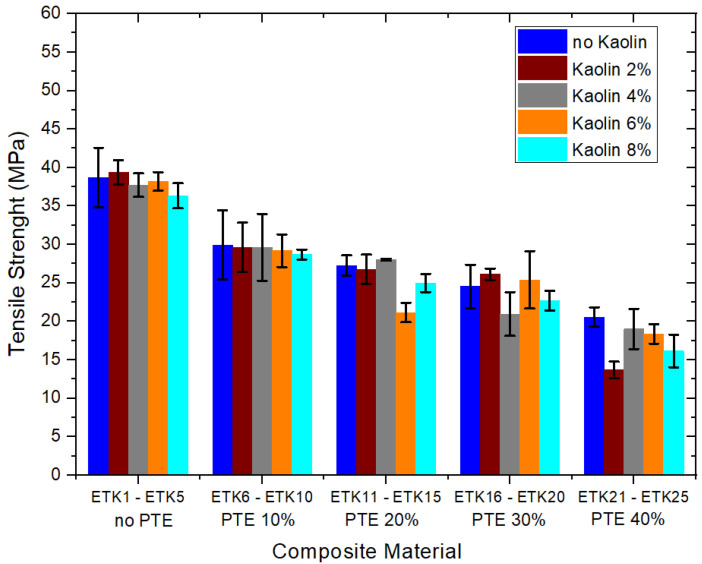
Results of the mechanical tests for tensile strength.

**Figure 13:**
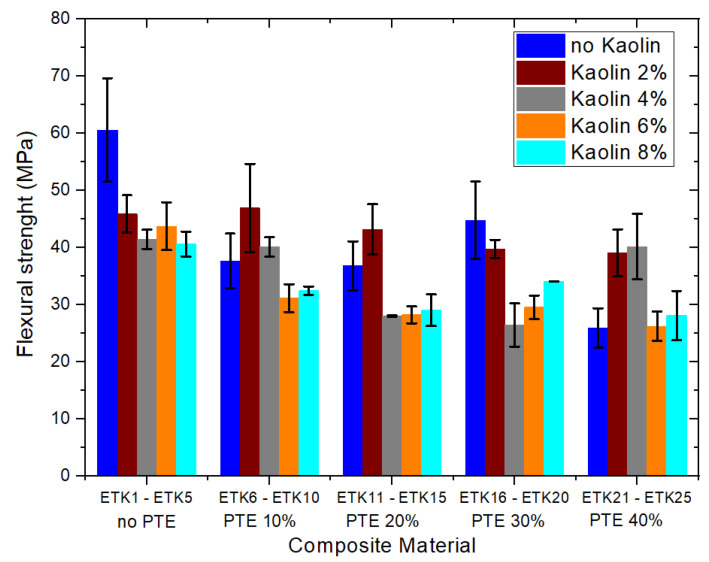
Results of the mechanical tests for flexural strength.

**Figure 14:**
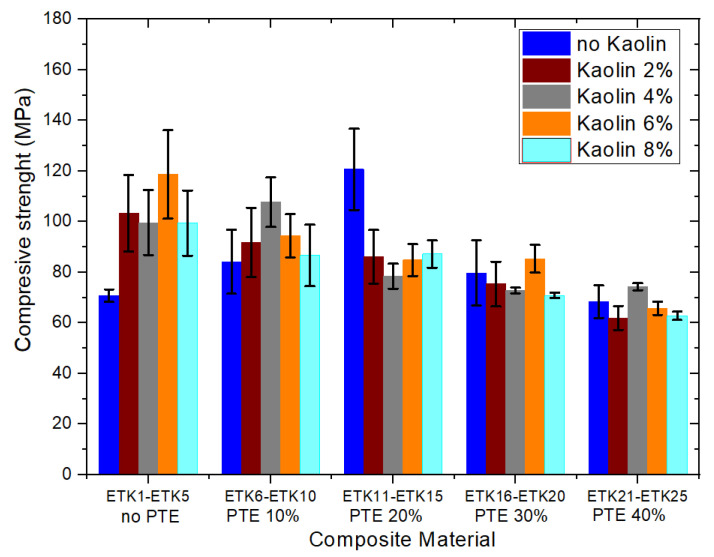
Results of the mechanical tests for compressive strength.

**Figure 15:**
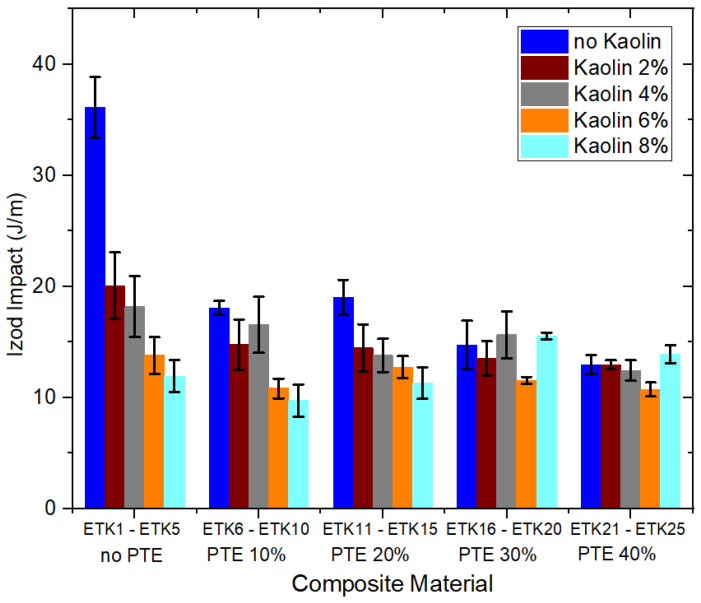
Results of the mechanical tests for Izod impact.

Figure 12, Figure 13, Figure 14 and Figure 15 present the results of the mechanical tests for tensile strength (Figure 12), flexural strength (Figure 13), compressive strength (Figure 14), and Izod impact of the samples developed for the current study (Figure 15). In the tensile test (Figure 12), the samples ETK2, ETK3, ETK4, and ETK5, with ER + kaolin without PTE, showed stability around 38 MPa, even with the increase in kaolin ranging from 2% to 8% by weight, which is a result that is about 100% superior to that presented in previous studies with recycled PP + PTE thermoplastic resin, as presented by Kieling et al. [20]. The PTE increment of 10% to 40% by weight in the composition without kaolin (samples ETK6, ETK11, ETK16, and ETK21) showed a significant decrease in tensile strength. The flexural test (Figure 13) showed a decrease from 61 MPa (sample ETK1) to approximately 26 MPa (samples ETK18, ETK11, and ETK24)—in other words, a reduction of 57%. The increase in kaolin from 2% to 8% in weight showed an average decrease of up to 50% in flexural strength for ER + PTE + kaolin composites. The best performance was in the ETK7 sample, with 47 MPa. For the compressive strength (Figure 14), the samples without PTE, with ER + kaolin, showed better performance, as can be seen in the samples ETK2, ETK3, ETK4, and ETK5, with emphasis on ETK11 (ER + 20% PTE) which had the best performance at around 120 MPa. For the Izod impact test (Figure 15), the compositions without PTE and with kaolin showed a reduction of 50%, on average, in resistance, and the addition of powdered tucumã did not improve impact resistance. The best performance was in the ETK6 (ER + 10% PTE) sample and the ETK11 (ER + 20% PTE) sample, which was between 18 J.m and 20 J.m.

Tucumã endocarp and kaolinite are abundant in the State of Amazonas and can be a sustainable alternative for the bioeconomy. Studies presenting organic particles of tucumã endocarp and inorganic clay minerals, which act together as fillers in polymer matrices, are still uncommon in the literature; this increases the importance of this work.

Composites with ER combined with kaolin (ETK2, ETK3, ETK4, and ETK5), without PTE, generally had better mechanical characteristics when compared with those with ER + PTE + kaolin, since the addition of kaolin leads to the loss of important mechanical properties. Despite these losses, composites resulting from ER + PTE (10% to 40%) + kaolin (2% and 4%), ETK7 to ETK10, and ETK12 to ETK15, presented significant potential for use in general industrial products.

## 4. Conclusions

The characterization of the powdered tucumã endocarp and kaolin from the State of Amazonas, as well as the composition epoxy resin + tucumã powder + kaolin, was carried out using FTIR, particle size analysis, and scanning electron microscopy and mechanical testing. The kaolin presented a significant clay portion (granulation below 2 μm) and pseudo-hexagonal structure in lamellar layers and the powdered tucumã had a polymorphic irregular rough structure. The results showed that composites with epoxy resin + powdered tucumã had mechanical characteristics that were superior to the composites with epoxy resin + kaolin and epoxy resin + powdered tucumã + kaolin. Tensile strength, flexural strength, compression tests, and impact tests were carried out with the aim of discovering which combination had the best mechanical characteristics. In this sense, in addition to the epoxy resin + powdered tucumã compounds, the epoxy resin + powdered tucumã (10% to 40% by weight) + kaolin (2% to 4% by weight) compounds stood out as promising composites for applications in general industry, especially in the civil construction industry for boards and coatings.

## Figures and Tables

**Table 1:** Identification of ER, PTE, and kaolin samples and compositions.

Sample	PTE	Kaolin
ETK1	0	0
ETK2	0	2
ETK3	0	4
ETK4	0	6
ETK5	0	8
ETK6	10	0
ETK7	10	2
ETK8	10	4
ETK9	10	6
ETK10	10	8
ETK11	20	0
ETK12	20	2
ETK13	20	4
ETK14	20	6
ETK15	20	8
ETK16	30	0
ETK17	30	2
ETK18	30	4
ETK19	30	6
ETK20	30	8
ETK21	40	0
ETK22	40	2
ETK23	40	4
ETK24	40	6
ETK25	40	8

## Data Availability

Not applicable.

## References

[1] Filizzola D.M., Santos T.D.S., de Miranda A.G., da Costa J.C.M., Nascimento N.R.D., dos Santos M.D., Bello R.H., del Pino G.G., Neto J.C.D.M. (2021). Annealing Effect on the Microstructure and Mechanical Properties of AA 5182 Aluminum Alloy. Mat. Res..

[2] Vieira A., de Macedo Neto J., de Miranda A., Verçosa L., Garcia del Pino G., Rodrigues R.D., Nascimento D. (2021). Influence of Thermal Treatment of Standardization in the Microstructure and Mechanical Properties of Sae 1035 Steel Used In Motorcycles. Engenharia de Materiais e Metalúrgica: Tudo à Sua Volta 2.

[3] Nagavally R.R. Composite materials—History, types, fabrication techniques, advantages, and applications. Proceedings of the 29th IRF International Conference.

[4] Botha N., Inglis H.M., Labuschagne F. Analysis of mechanical property degradation in polymer nanoclay composites. Proceedings of the Third International Conference on Composites, Biocomposites and Nanocomposites.

[5] Awwad K.E., Yousif B., Mostafa A., Alajarmeh O., Zeng X. (2022). Tribological and mechanical performances of newly developed eco-epoxy composites incorporating flax fibres and graphene nanoplatelets. J. Reinf. Plast. Compos..

[6] Krauklis A.E., Karl C.W., Gagani A.I., Jørgensen J.K. (2021). Composite Material Recycling Technology—State-of-the-Art and Sustainable Development for the 2020s. J. Compos. Sci..

[7] Post W., Susa A., Blaauw R., Molenveld K., Knoop R.J.I. (2020). A Review on the Potential and Limitations of Recyclable Thermosets for Structural Applications. Polym. Rev..

[8] Mishnaevsky L. (2021). Sustainable End-of-Life Management of Wind Turbine Blades: Overview of Current and Coming Solutions. Materials.

[9] Effect of Clay Modification on the Morphological, Mechanical, and Thermal Properties of Polyamide 6/polypropylene/montmorillonite Nanocomposites-Kusmono-2010-Polymer Composites-Wiley Online Library. https://4spepublications.onlinelibrary.wiley.com/doi/full/10.1002/pc.20902.

[10] Guo F., Aryana S., Han Y., Jiao Y. (2018). A Review of the Synthesis and Applications of Polymer–Nanoclay Composites. Appl. Sci..

[11] Sarges R.R., Nogueira A.C.R., Frota C.A., da Silva C.L. (2010). Depósitos Argilosos Cenozóicos do Estado do Amazonas: Utilização Como Agregados de Argilas Calcinadas Para Pavimentações Na Região Amazônica. Braz. Geogr. J..

[12] Venkatesan N., Bhaskar G.B., Rajesh S., Pazhanivel K., Sagadevan S. (2017). Effect of Cloisite 30B nanoclay on the mechanical properties of HDPE nanocomposites. Mater. Test..

[13] Rao G.S., Shankar H.R. (2018). Effect of Nano Clay inclusions on Mechanical Properties of Thermoplastics. Eur. J. Eng. Sci. Technol..

[14] Deka B.K., Maji T.K. (2013). Effect of TiO_2_ and nanoclay on the properties of wood polymer nanocomposite. Polym. Bull..

[15] García del Pino G., Kieling A.C., Bezazi A., Boumediri H., Rolim de Souza J.F., Valenzuela Díaz F., Valin Rivera J.L., Dehaini J., Panzera T.H. (2020). Hybrid Polyester Composites Reinforced with Curauá Fibres and Nanoclays. Fibers Polym..

[16] Robledo-Ortíz J.R., del Campo A.M., López-Naranjo E.J., Arellano M., Jasso-Gastinel C.F., González-Núñez R.L., Pérez-Fonseca A.A. (2019). Effect of low nanoclay content on the physico-mechanical properties of poly(lactic acid) nanocomposites. Polym. Polym. Compos..

[17] Islam S.M., Hamdan S., Talib Z.A., Ahmed A.S., Rahman M.R. (2012). Tropical wood polymer nanocomposite (WPNC): The impact of nanoclay on dynamic mechanical thermal properties. Compos. Sci. Technol..

[18] Pickering K.L., Efendy M.G.A., Le T.M. (2016). A review of recent developments in natural fibre composites and their mechanical performance. Compos. Part A Appl. Sci. Manuf..

[19] Khelifa H., Bezazi A., Boumediri H., del Pino G.G., Reis P.N.B., Scarpa F., Dufresne A. (2021). Mechanical characterization of mortar reinforced by date palm mesh fibers: Experimental and statistical analysis. Constr. Build. Mater..

[20] Kieling A.C., Santana G.P., Santos M.C.D., Neto J.C.D.M., Pino G.G.D., Santos M.D.D., Duvoisin S., Panzera T.H. (2021). Wood-plastic Composite Based on Recycled Polypropylene and Amazonian Tucumã (Astrocaryum aculeatum) Endocarp Waste. Fibers Polym..

[21] Richely E., Bourmaud A., Placet V., Guessasma S., Beaugrand J. (2022). A critical review of the ultrastructure, mechanics and modelling of flax fibres and their defects. Prog. Mater. Sci..

[22] Baley C., Gomina M., Breard J., Bourmaud A., Davies P. (2020). Variability of mechanical properties of flax fibres for composite reinforcement. A review. Ind. Crops Prod..

[23] Kieling A.C., Santana G.P., Santos M.C.D., de Cassia Castro Jaqtinon H., Monteiro C.C.P. (2019). Cadeia do tucumã comercializado em Manaus-AM. Sci. Amazon..

[24] (2020). Standard Test Method for Particle Size Distribution of Catalytic Materials by Laser Light Scattering.

[25] Savazzini-Reis A., Savazzini-Reis V.P., Valenzuela Díaz F.R. Caracterização e propriedades cerâmicas de argilas usadas em cerâmica vermelha no Estado do Espírito Santo. Proceedings of the 22° CBECiMat—Congresso Brasileiro de Engenharia e Ciência dos Materiais.

[26] Kieling A.C., Neto J.C.M., Pino G.G., Dantas-dos Santos M., Santana G.P., Da-Silva R.J., Panzera T.H., Valenzuela M.S., Diáz F.R.V. (2023). Epoxy-based hybrid composites reinforced with Amazonian Tucumã endocarp and kaolin: A statistical approach to mechanical Properties. Materialia.

[27] (2021). Standard Practice for General Techniques for Obtaining Infrared Spectra for Qualitative Analysis.

[28] Van Soest P.J. (1963). Use of detergents in the analysis of fibrous feeds. 2. A rapid method for the determination of fiber and lignin. J. Assoc. Off. Agric. Chem..

[29] (2017). Standard Practice for Scanning Electron Microscope Beam Size Characterization.

[30] (2014). American Society for Testing and Materials Standard Test Method for Tensile Properties of Plastics.

[31] (2006). Standard Test Methods for Flexural Properties of Unreinforced and Reinforced Plastics and Electrical Insulating Materials.

[32] (2002). Standard Test Method for Compressive Properties of Rigid Plastics.

[33] (2002). Standard Test Methods for Determining the Izod Pendulum Impact Resistance of Plastics.

[34] Zhang W., Yin L., Zhao M., Tan Z., Li G. (2021). Rapid and non-destructive quality verification of epoxy resin product using ATR-FTIR spectroscopy coupled with chemometric methods. Microchem. J..

[35] Otieno S.O., Kengara F.O., Kemmegne-Mbouguen J.C., Langmi H.W., Kowenje C.B.O., Mokaya R. (2019). The effects of metakaolinization and fused-metakaolinization on zeolites synthesized from quartz rich natural clays. Microporous Mesoporous Mater..

[36] Deju R., Mazilu C., Stanculescu I., Tuca C. (2020). Fourier Transform Infrared Spectroscopic Characterization of Thermal Treated Kaolin. Rom. Rep. Phys..

